# IGF2/IGF1R Signaling as a Therapeutic Target in MYB-Positive Adenoid Cystic Carcinomas and Other Fusion Gene-Driven Tumors

**DOI:** 10.3390/cells8080913

**Published:** 2019-08-16

**Authors:** Mattias K. Andersson, Pierre Åman, Göran Stenman

**Affiliations:** Sahlgrenska Cancer Center, Department of Pathology, University of Gothenburg, 405 30 Gothenburg, Sweden

**Keywords:** IGF signaling, IGF2, IGF1R, gene fusions, MYB, adenoid cystic carcinoma, salivary gland tumor, carcinoma, FET oncogenes, sarcoma

## Abstract

Chromosome rearrangements resulting in pathogenetically important gene fusions are a common feature of many cancers. They are often potent oncogenic drivers and have key functions in central cellular processes and pathways and encode transcription factors, transcriptional co-regulators, growth factor receptors, tyrosine kinases, and chromatin modifiers. In addition to being useful diagnostic biomarkers, they are also targets for development of new molecularly targeted therapies. Studies in recent decades have shown that several oncogenic gene fusions interact with the insulin-like growth factor (IGF) signaling pathway. For example, the *MYB–NFIB* fusion in adenoid cystic carcinoma is regulated by IGF1R through an autocrine loop, and IGF1R is a downstream target of the EWSR1–WT1 and PAX3–FKHR fusions in desmoplastic small round cell tumors and alveolar rhabdomyosarcoma, respectively. Here, we will discuss the mechanisms behind the interactions between oncogenic gene fusions and the IGF signaling pathway. We will also discuss the role of therapeutic inhibition of IGF1R in fusion gene driven malignancies.

## 1. Introduction

Fusion genes are potent oncogenes generated by chromosome rearrangements, in particular translocations. The first gene fusions in cancer were discovered in the early 1980s [1,2,3,4] and since then numerous fusions have been identified, many of which are specific for a certain type of neoplasm [4]. Large-scale genomic sequencing together with comprehensive functional studies have confirmed that gene fusions are key oncogenic drivers not only in myelo- and lymphoproliferative disorders but also in sarcomas, carcinomas, and benign mesenchymal and epithelial tumors [3,5,6]. Gene fusions have significantly contributed to a refined subclassification of both leukemias and certain solid tumors and are now used in routine clinical practice as diagnostic biomarkers [2,3,5,6]. They may for example encode transcription factors, transcriptional co-regulators, growth factor receptors, tyrosine kinases, and chromatin modifiers. Importantly, recent studies have demonstrated that the chimeric proteins encoded by these fusions can serve as targets for the development of new cancer therapies. The prototype example being the *BCR*–*ABL1* fusion in chronic myeloid leukemia, which encodes a fusion protein with deregulated tyrosine kinase activity [7,8]. Other examples include the *PML*–*RARA* fusion in acute promyelocytic leukemia [9], the *COL1A1*–*PDGFRB* fusion in dermatofibrosarcoma protuberans [10,11], and the *EML4*–*ALK* fusion in non-small-cell lung cancer [12,13]. For these and other fusion gene driven cancers, targeted therapies have been developed that significantly improve the outcome for affected patients.

Studies in recent decades have shown that several oncogenic gene fusions interact with insulin-like growth factor (IGF) signaling at different levels [14,15]. IGF signaling is an ancient evolutionary conserved mechanism that regulates critical cellular processes such as cell proliferation and survival [16]. In humans, the IGF system is comprised of the two ligands IGF1 and IGF2, their target tyrosine kinase receptors, IGF1 receptor (IGF1R) and the insulin receptor (INSR), as well as the IGF2 receptor (IGF2R) and IGF-binding proteins (IGFBPs) that regulate IGF ligand availability. In this review, we will discuss the mechanisms behind some of the interactions between oncogenic gene fusions and IGF signaling with a focus on the role of IGF1R.

## 2. The *MYB–NFIB* Gene Fusion in Adenoid Cystic Carcinoma

Adenoid cystic carcinoma (ACC) is a clinically challenging tumor with a high rate of recurrence and distant metastases [17]. ACCs most often arise in the head and neck but may also originate in the breast, lung, skin, and other sites. ACCs in the head and neck (mainly salivary glands) usually have a poor long-term prognosis and there is no effective systemic treatment available for patients with inoperable tumors [18,19,20]. We have previously shown that the genomic hallmark of ACC is a t(6;9)(q23;p23) translocation [21], which results in a fusion of the two transcription factor genes *MYB* and *NFIB* (Table 1) [22]. 

*MYB* encodes a master transcriptional regulator that is highly expressed in hematopoetic stem/progenitor cells and in colonic stem cells as well as in leukemias and certain carcinomas [23]. The regulation of *MYB* expression is complex and two separate promoters have been identified that are regulated by different transcription factors. Moreover, the expression of *MYB* mRNA is controlled by an intricate transcriptional pausing mechanism in the first intron and by miRNAs binding to the 3’ UTR. The MYB protein has an N-terminal DNA-binding domain and a central transactivation domain. The latter binds transcriptional co-activators that regulate the expression of target genes involved in cell cycle control, differentiation, and cell survival. MYB activity is tightly regulated through an autoinhibitory negative regulatory domain in the C-terminus that is abrogated in truncated oncogenic variants of the protein. Recent studies have shown that MYB binds super-enhancers [24,25], i.e., chromosomal regions with a high degree of H3K27 acetylation.

NFIB is a member of the Nuclear Factor I (NFI) family of DNA-binding transcription factors [26]. It is a master regulator of differentiation in multiple organs and is expressed in most human tissues. *NFIB* is regulated in a cell-type dependent manner and has a long 3’-UTR with binding sites for regulatory miRNAs [27]. Depending on the cellular context, NFIB can either activate or repress target genes [26]. *NFIB* can also act both as an oncogene and a tumor suppressor gene depending on the tumor type [27]. Similar to MYB, NFIB can bind super-enhancers [28]. Notably, super-enhancer elements have been identified in the 3’-part of *NFIB* and its flanking sequences [29].

The predicted MYB–NFIB fusion proteins contain the DNA-binding and transactivation domains of MYB fused to the C-terminal end of NFIB. The minimal common region of NFIB fused to MYB is only five amino acids (SWYLG) encoded by the last exon [22,30]. Recent studies have shown that MYB–NFIB fusion promotes tumor cell proliferation in ACC by regulating genes involved in the cell cycle, RNA processing, and DNA-repair [31]. In particular, MYB–NFIB was shown to regulate anchorage-independent growth of ACC stem/progenitor cells, highlighting the fusion as a potential therapeutic target. However, oncogenic transcription factors are notoriously difficult to target and there are few existing cancer drugs with this mechanism of action [32,33]. Except for a small subset of tumors with NOTCH-pathway mutations, there are no other major genomic alterations in ACC that may be targeted therapeutically [34]. Moreover, ACC shows an overall low mutational burden that is associated with a poor response to immunotherapy in other malignancies [35]. Therefore, new treatment strategies are needed for ACC patients.

### 2.1. The MYB–NFIB Fusion Is Regulated by IGF1R in an AKT-Dependent Manner

**Figure 1:**
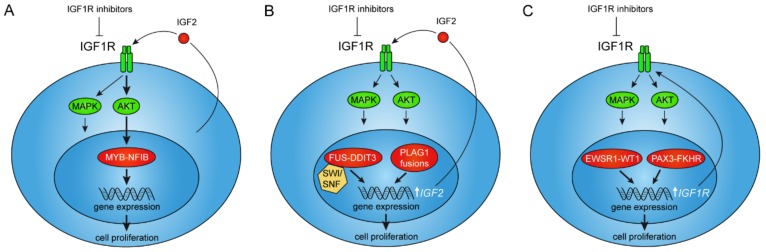
Interactions between oncogenic gene fusions and IGF2/IGF1R signaling. (**A**) The MYB–NFIB fusion is positively regulated by AKT-dependent IGF1R signaling as a result of autocrine IGF2-stimulation in adenoid cystic carcinoma. (**B**) PLAG1 and FUS–DDIT3 fusions directly regulate expression of the *IGF2*-gene leading to autocrine stimulation of IGF1R signaling in pleomorphic adenoma, hepatoblastoma, and myxoid liposarcoma. (**C**) The EWSR1–WT1 and PAX3–FKHR fusions regulate transcription of the *IGF1R* gene leading to overexpression of its encoded tyrosine kinase receptor in desmoplastic small round cell tumor and alveolar rhadomyosarcoma, respectively.

Until recently, little was known about receptor tyrosine kinase (RTK) signaling in ACC and clinical studies of ACC patients had shown poor response to targeted therapies [20]. Using MYB–NFIB positive, short-term cultured human ACC cells, Andersson and co-workers recently demonstrated that IGF1R, INSR, MET, and EGFR are consistently activated in ACCs [31]. They also showed that these receptors drive proliferation of ACC cells through AKT and MAPK signaling. The combined inhibition of IGF1R/INSR/MET/EGFR caused a synergistic decrease in ACC cell proliferation and in the growth of ACC patient-derived xenograft (PDX) models. The treatment also induced differentiation both in vitro and in vivo and unexpectedly led to the downregulation of *MYB–NFIB* expression. Further studies revealed that *MYB–NFIB* expression in ACC is regulated by IGF1R in an AKT-dependent manner (Figure 1A) [31]. This effect was fusion gene specific since the wild-type, non-fused *MYB* allele was unaffected by treatment with IGF1R inhibitors. Moreover, ACC was shown to specifically overexpress IGF2, suggesting a feedback mechanism between MYB–NFIB and IGF2. These findings imply that IGF2 is the endogenous factor regulating IGF signaling in ACC, and hence MYB–NFIB expression, through autocrine stimulation (Figure 1A). Notably, pharmacologic inhibition of IGF1R was shown to reverse the oncogenic transcriptional program induced by MYB–NFIB in ACC cells. Taken together, these results demonstrate that the IGF2–IGF1R–MYB–NFIB axis is a key target for therapy in ACC. The study by Andersson et al. uncovered a new strategy to target an oncogenic transcriptional master regulator and provide new important insights into the role of IGF1R signaling in fusion gene driven malignancies [31].

### 2.2. Treatment of ACC Patients with IGF1R Inhibitors

The importance of IGF1R signaling in ACC is further supported by clinical case studies using humanized monoclonal antibodies against IGF1R. Morelli et al. reported on a 29-year-old woman with ACC who was resistant to standard of care treatments [36]. Screening of a personalized PDX model with a selection of anticancer drugs revealed that one of the most effective agents was the IGF1R antibody figitumumab which induced significant tumor growth inhibition (76%) compared to untreated controls. Subsequent treatment of the patient with figitumumab resulted in a minor response that lasted for 6 months, after which the disease progressed, and the patient was taken off the study. Calvo et al. reported a partial response lasting for over 1.5 years in an ACC patient treated with figitumumab in combination with the EGFR inhibitor dacomitinib and stable disease as best overall response in another ACC patient receiving the same treatment [37]. Finally, Mahadevan et al. reported stable disease for over a year in an ACC patient treated with the IGF1R antibody R1507 in combination with sorafenib [38]. R1507 was well tolerated and did not interfere with glucose metabolism. Taken together, data from both preclinical and clinical studies provide strong evidence that IGF1R signaling is crucial for ACC growth and progression, and that the IGF2–IGF1R–MYB–NFIB axis is a key target for therapy in ACC.

## 3. IGF Signaling as a Therapeutic Target in Tumors with Activation of the *PLAG1* Oncogene

Pleomorphic adenomas are benign tumors originating from the major and minor salivary glands [39]. They may also occur in other anatomical locations, including the breast, lung, skin, and female genital tract. Although pleomorphic adenomas are benign, they may cause clinical problems due to their propensity for recurrence and malignant transformation to carcinoma ex-pleomorphic adenoma. The latter is usually a highly malignant tumor.

The genomic hallmarks of pleomorphic adenomas are translocations targeting the *PLAG1* (pleomorphic adenoma gene 1) and *HMGA2* (high mobility group A2) oncogenes [40,41]. *PLAG1* was originally identified as the target gene of a recurrent t(3;8)(p21;q12) translocation in pleomorphic adenomas which results in promoter swapping between the ubiquitously expressed *CTNNB1* (β-catenin) gene and the developmentally regulated *PLAG1* gene, leading to activation of *PLAG1* expression (Table 1) [40,42,43]. PLAG1 is a transcription factor with seven canonical C2H2 zinc finger domains in the N-terminus and a serine-rich transcriptional activation domain in the C-terminus [40,44]. The gene is highly expressed in fetal kidney, liver, and lung, whereas the expression is low or absent in adult tissues [40]. In addition to the *CTNNB1–PLAG1* fusion, there are at least eight additional known *PLAG1* fusions in pleomorphic adenomas and myoepithelial carcinoma ex-pleomorphic adenomas that lead to ectopic overexpression of wild-type PLAG1 proteins [6,45]. Notably, previous studies have shown that *PLAG1* is also involved in similar gene fusions with other partner genes in lipoblastomas [46,47,48]. In addition, *PLAG1* is amplified and overexpressed in hepatoblastomas associated with poor prognosis [49,50]. 

PLAG1 exerts its oncogenic activities through the expression of its target genes involved in for example growth control, cell proliferation, apoptosis, and transcriptional regulation [51,52]. Studies of knockout mice have confirmed a major role for PLAG1 in growth control; PLAG1 deficiency causes growth retardation in mice [53]. Known PLAG1 target genes include for example *IGF2*, *CYTL1*, *CRABP2*, *SMARCD3*, *BCL2*, *CDKN1C*, *EFNB1*, and *TSPAN4* [52]. Of these, most attention has been focused on the maternally imprinted *IGF2* gene. Notably, PLAG1 binds the embryonic P3 promoter of IGF2 and activate its expression in pleomorphic adenomas overexpressing PLAG1 (Figure 1B) [54]. Similar to PLAG1, IGF2 is necessary for normal embryonic growth [55]. PLAG1 also transactivates transcription from the embryonic IGF2 P3 promoter in hepatoblastomas, leading to overexpression of IGF2 [49]. Taken together, these observations suggest that the oncogenic activities of PLAG1 is at least partly mediated by IGF2 signaling and that IGF2 signaling is a potential therapeutic target in pleomorphic adenoma and hepatoblastoma and possibly also in lipoblastoma. However, initial studies have shown poor efficacy after treatment with single-agent cixutumumab, an IGF1R inhibitor, in a phase II trial including 10 patients with refractory hepatoblastomas [56]. Additional studies will be needed to fully evaluate the efficacy of single or combined IGF inhibition in these neoplasms. 

## 4. IGF1R Signaling Is Required for *ETV6–NTRK3*-Mediated Tumorigenesis

The *ETV6–NTRK3* gene fusion was originally identified in congenital fibrosarcomas with t(12;15)(p13;q25) translocations (Table 1) [57]. Subsequent studies demonstrated that the *ETV6–NTRK3* fusion, in contrast to many other gene fusions, may occur in a variety of neoplasms in different anatomical locations, including secretory carcinomas of the breast and salivary glands [58,59], mesoblastic nephromas [60], acute leukemias [61], pediatric non-brainstem high-grade gliomas [62], papillary thyroid carcinomas [63], colorectal carcinomas [64], and inflammatory myofibroblastic tumors (Table 1) [65]. The fusion links the dimerization domain of the ETV6 transcription factor to the tyrosine kinase domain of NTRK3 (neurotrophin-3 receptor) leading to constitutive activation of the NTRK3 kinase [66]. The identification of TRK fusions in a variety of cancers have prompted the development of selective TRK inhibitors, such as larotrectinib or entrectinib, which have shown high response rates (≈75%) across children and adults with TRK-fusion cancers [67,68]. However, despite these promising results, many patients with advanced-stage TRK-fusion cancers eventually become resistant to TRK inhibition [67].

An alternative target for treatment of TRK-fusion cancers was identified by Sorensen and co-workers who showed that the ETV6–NTRK3 fusion requires an intact IGF1R–PI3K–AKT axis for neoplastic transformation [69,70], and that IGF1R inhibitors can block ETV6–NTRK3-mediated tumorigenesis in vivo [66,70]. Recent studies have also shown that IGF1R inhibition induces rapid ubiquitylation and degradation of the ETV6–NTRK3 fusion protein through a proteasome-dependent mechanism mediated by the E3 ubiquitin ligase KPC1 [71]. These results highlight a novel strategy for targeting TRK-fusion cancers. 

## 5. The FET Fusion Oncogenes in Sarcomas and Leukemias

**Figure 2:**
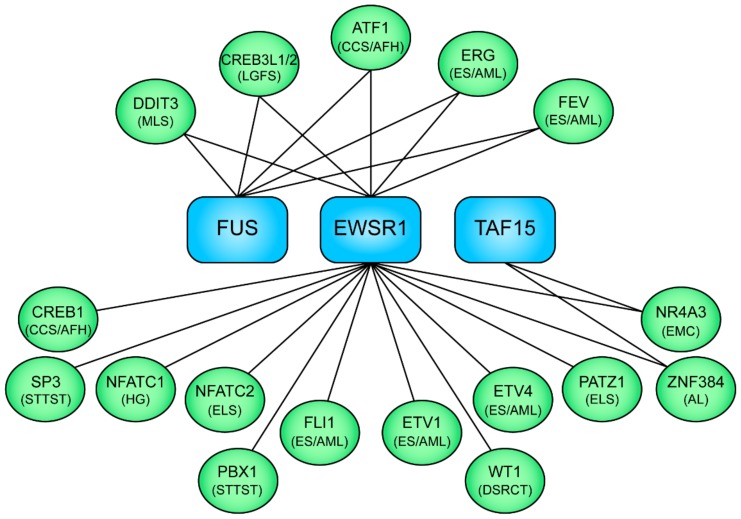
Schematic illustration of selected examples of the FET family of fusion oncoproteins and their associated neoplasms. The N-terminal fusion partners EWSR1, FUS, and TAF15 are shown in blue boxes, the C-terminal transcription factor fusion partners in green ovals, and the associated neoplasms in parenthesis. MLS, myxoid liposarcoma; LGFS, low-grade fibromyxoid sarcoma; CCS, clear cell sarcoma; AFH, angiomatoid fibrous histiocytoma; ES, Ewing sarcoma; AML, acute myeloid leukemia; STTST, soft tissue tumor special type; HG, hemangioma (bone); ELS, Ewing-like sarcoma; DSRCT, desmoplastic small round cell tumor; EMC, extraskeletal myxoid chondrosarcoma; AL, acute leukemia.

Over 20 different types of sarcomas and leukemias contain fusion oncogenes with the FET genes *FUS, EWSR1*, and *TAF15* as 5’-partners and different transcription factor genes as 3’-partners [4,72,73,74] (Figure 2). The wild-type FET genes encode homologous RNA-binding proteins involved in gene regulation at different levels [75,76]. The FET fusion oncogenes are genomic hallmarks of a subset of sarcomas and are considered as major oncogenic drivers with few, or in some cases, no other known driver mutations [73,77,78]. The FET fusion oncoproteins regulate central cellular processes such as cell fate, differentiation, and proliferation. 

The FET fusion oncoproteins consist of the N-terminal domains (NTDs) of the FET proteins linked to the DNA-binding domains of the transcription factor partners (Figure 2). They act as aberrant transcription factors with the FET NTDs as strong transactivators [79,80,81,82,83]. Forced expression or silencing of FET fusions affect a large number of genes and change the epigenetic landscape [82,84,85,86]. Recently, major fractions of endogenously expressed FET fusion proteins were shown to bind the SWI–SNF chromatin-remodeling complex in sarcoma cells (Figure 1B) [86]. The SWI–SNF complex is a large multi-subunit complex that controls gene expression by repositioning of nucleosomes and suppressive polycomb complexes [87,88]. The importance of the SWI–SNF complex in tumorigenesis is also illustrated by the numerous mutations affecting SWI–SNF components in various cancers [89]. The interaction between FET oncoproteins and the SWI–SNF complex provides new mechanistic insights into the oncogenic activities of FET fusions, and further underscores the importance of dysfunctional SWI–SNF complexes in cancer. 

The aberrant transcription factor activities involving SWI–SNF interactions of the FET oncogenes are likely responsible for the contradictory gene marker expression profiles seen in FET fusion cancers [82,86,87]. In at least three of these tumor types, members of the IGF signaling pathway are involved. The FET oncogene tumors are thought to originate from primitive mesenchymal or hematopoetic stem/progenitor cells [73,77,83,90], and origin from such primitive, poorly differentiated cells may explain why the IGF signaling pathway is activated in these tumors. 

### 5.1. IGF/IGF1R Signaling in FET Fusion Oncogene Sarcomas

Ewing sarcoma (ES) is an aggressive bone and more rarely soft tissue tumor preferentially occurring in young individuals [91]. ES is characterized by a plethora of fusions between EWSR1 or FUS and different ETS-binding transcription factors (Figure 2) [91]. Major fractions of the ES fusion oncoproteins bind the SWI–SNF complex [86] and the recruitment of this complex results in activation of ETS sites across the genome [87]. This may partially explain the puzzling mixed mesenchymal and neural gene marker expression profile in ES cells [91]. Many marker genes are the direct targets of fusion oncoproteins, including I*GFBP3* that is repressed by the ES prototype fusion EWSR1–FLI1 (Table 1) [92]. The expression of IGF1R and IGF1 in ES cells is part of an autocrine loop and blockage of this loop by IGFBP3 halts the proliferation of cultured ES cells. Moreover, blocking of the loop with an IGF1R monoclonal antibody inhibits proliferation and increases apoptosis of ES cells in vitro and in vivo [93]. IGF1R expression is also necessary for neoplastic transformation of normal cells by the EWSR1–FLI1 fusion [94]. 

Desmoplastic small round cell tumors (DSRCT) are highly aggressive lesions preferentially occurring in children and young adult males [95]. They are characterized by *EWSR1–WT1* gene fusions (Table 1). Notably, the EWSR1–WT1 fusion protein, directly binds to and upregulates the expression of *IGF1R* (Figure 1C) [96]. This is in contrast to the suppressive effect on *IGF1R* of the wildtype WT1 protein [97]. The EWSR1–WT1-mediated upregulation of IGF1R leads to an increased sensitivity to receptor ligands resulting in growth stimulation [98].

Myxoid liposarcomas (MLS) most commonly occur in skeletal muscles, typically in young to middle age individuals. They can often be successfully treated with radical surgery, sometimes in combination with radio- or chemotherapy [99]. MLS are characterized by *FUS–DDIT3* or *EWSR1–DDIT3* gene fusions (Table 1) [100,101,102,103]. However, in 10–15% of the cases, secondary mutations occur in *PTEN* or *PIK3CA* [104]. These mutations are associated with a small round cell phenotype, metastases, and poor prognosis [99,104]. Similar to ES and DSRCT, MLS overexpress IGF1R [105]. MLS cells also express IGF2 and forced expression of FUS–DDIT3 induces expression of IGF2 (Figure 1B). Treatment with IGF1R inhibitors lead to decreased proliferation of MLS cells, indicating a growth stimulatory effect of the IGF2–IGF1R autocrine loop [105]. However, no clinical trials targeting the IGF signaling pathway have so far been reported in MLS patients.

Although so far only a few of the FET oncogene tumors have been shown to express IGF ligands and/or their receptors, their similar cellular origin and molecular pathogenesis, suggest that also other members of the FET family of tumors may interact with the IGF signaling pathway. 

### 5.2. Anti-IGF Treatment of FET Fusion Oncogene Sarcomas

The discovery of aberrant IGF1R signaling in FET gene fusion-associated sarcomas has inspired many trials to exploit this pathway for therapeutic purposes. In a phase I/II study using the IGF1R antibody figitumumab, 15 of 106 ES patients showed a partial response and 25 had stable disease [106]. The median overall survival was 8.9 months. A strong association between pretreatment serum IGF1 and survival benefit was found. It was concluded that figitumumab had modest activity as single agent in advanced ES. In a phase II study of 115 ES patients, treatment with the IGF1R antibody R1507 resulted in one complete response, 10 partial responses, and 18 patients with stable disease [107]. Another anti-IGF1R antibody, ganitumab, was used in a phase II study of 22 ES and 16 DSRCT patients with relapsed or refractory disease [108]. Of 35 patients assessed for response, two had partial responses and 17 had stable disease; four patients had stable disease for over 24 weeks. There was no apparent relationship between tumor response and the type of FET oncogene. In a fourth phase II study, including 115 ES patients treated with the humanized anti-IGF1R antibody robatumumab, PET-CT revealed six patients with partial response, 23 with stable disease, and 55 with progression of disease after two months [109]. The median overall survival was 6.9 months. Six patients with metastatic ES showed clinical response and remained healthy after receiving 25–115 doses of robatumumab with remissions of over 4 years duration.

A major problem with IGF1R inhibition is the rapid development of resistance and thus short-duration clinical response [16]. Resistance to agents targeting IGF1R is likely intrinsically associated with the complex, redundant interactions that characterize the IGF signaling pathway. Further trials should therefore focus on combinations of anti-IGF1R antibodies and other drugs. However, treatment with anti-IGF1R antibodies in combination with mTOR inhibitors gave no improvements compared to single treatment with anti-IGF1R in patients with ES and DSRCT [110,111,112].

Taken together, inhibition of the IGF1R pathway has proved more promising in the FET oncogene associated tumors ES and DSRCT than in many other tumor types. However, the limited number of responding patients and the short-duration clinical responses remain major obstacles that need to be overcome. Future studies should focus on identifying biomarkers that can predict responders and non-responders to IGF1R inhibition. 

## 6. IGF1R Is a Downstream Target of the PAX3–FKHR Fusion in Rhabdomyosarcoma

There are two main subtypes of rhabdomyosarcomas, embryonal (ERMS) and alveolar (ARMS) rhabdomyosarcomas. ERMS usually affects children, whereas ARMS typically affect all age groups equally. ARMS most often occur in the extremities. In contrast to ERMS, ARMS are characterized by a recurrent t(2;13) translocation resulting in a fusion between the *PAX3* and *FKHR* (*FOXO1*) genes (Table 1) [113]. *PAX3* encodes a transcription factor with an N-terminal DNA-binding domain (paired box domain) and *FKHR* encodes a forkhead transcription factor. The predicted PAX3–FKHR fusion oncoprotein contains an intact PAX3 DNA-binding domain linked to the bisected FKHR DNA-binding and transcriptional activation domains [114]. The PAX3–FKHR fusion protein is a more potent transcriptional activator than PAX3 alone [115]. Notably, IGF1R has been shown to be a direct downstream target of the PAX3–FKHR fusion and thereby also an interesting therapeutic target (Figure 1C) [116,117]. However, early clinical trials with IGF1R inhibitors have shown modest efficacy in rhabdomyosarcoma patients [118]. In addition, constitutive downregulation of IGFBP2 has been suggested as a mechanism of acquired resistance to IGF1R antibody therapy in ARMS [119]. 

## 7. Gene Fusions Involving *IGF1R*

Recent studies using RNA-seq and whole genome sequencing have generated a dramatic increase in the number of genes involved in gene fusions in human neoplasms [4,5]. Interestingly, there are now also single cases of breast and lung cancers, ALK-negative inflammatory myofibroblastic tumors (IMTs), and gastrointestinal stromal tumors (GISTs) described with chimeric fusions in which *IGF1R* serves as the 3’-partner gene [4,120,121]. The fusions are likely to be functional since they retain both the transmembrane and tyrosine kinase domains of IGF1R. Whether any of these fusions are recurrent is currently unknown. The identification of targetable chimeric fusions in single cases of for example *FN1–IGF1R* positive IMT and *NBPF1–IGF1R* positive GIST (Table 1) emphasize the need for personalized genetic profiling to determine the optimal treatment regimens for these patients. 

## 8. Concluding Remarks

The number of oncogenic gene fusions discovered in human cancer is steadily increasing due to large-scale, next-generation sequencing initiatives of human cancers, in particular carcinomas. Many gene fusions are potent oncogenic drivers with key functions in central cellular processes and pathways as illustrated in this review. Surprisingly, many gene fusions are also associated with aberrant IGF signaling. For example, the *MYB–NFIB* fusion in ACC is regulated by IGF1R through an autocrine loop, and IGF1R is a direct downstream target of the EWSR1–WT1 and PAX3–FKHR fusions in DSRCT and ARMS, respectively. These and other findings have thus identified IGF1R as a promising therapeutic target in different fusion gene driven neoplasms. However, so far, clinical trials with IGF1R inhibitors have only shown modest efficacy. Future studies need to focus on evaluating IGF1R inhibitors in combination therapies or as an adjunct to conventional chemotherapy and to find new biomarkers that can identify patients who will benefit from IGF1R inhibition.

## Figures and Tables

**Table 1:** Examples of chromosome translocations and gene fusions interacting with components of the insulin-like growth factor (IGF) signaling pathway.

Tumor Type	Chromosome Translocation	Gene Fusion
Adenoid cystic carcinoma	t(6;9)(q23;p23) t(8;9)(q13.1;p23)	*MYB–NFIB* *MYBL1–NFIB*
Pleomorphic adenoma	t(3;8)(p21;q12)	*CTNNB1–PLAG1*
Congenital fibrosarcoma Secretory carcinoma (breast and salivary gland) Mesoblastic nephroma Acute leukemia Pediatric non-brainstem high-grade glioma Papillary thyroid carcinoma Colorectal carcinoma Inflammatory myofibroblastic tumor	t(12;15)(p13;q25)	*ETV6–NTRK3*
Ewing sarcoma	t(11;22)(q24;q12)	*EWSR1–FLI1*
Desmoplastic small round cell tumor	t(11;22)(p13;q12)	*EWSR1–WT1*
Myxoid liposarcoma	t(12;16)(q13;p11) t(12;22)(q13;q12)	*FUS–DDIT3* *EWSR1–DDIT3*
Alveolar rhabdomyosarcoma	t(2;13)(q35;q14)	*PAX3–FKHR*
Inflammatory myofibroblastic tumor	t(2;15)(q35;q26.3)	*FN1–IGF1R*
Gastrointestinal stromal tumor	t(1;15)(p36.13;q26.3)	*NBPF1–IGF1R*
